# Some General Aspects of Poisons—II

**Published:** 1909-08-14

**Authors:** 


### Medico-Legal Points.

### SOME GENERAL ASPECTS OF POISONS—II.

A person is supposed to be poisoned if, being in perfect health, he is attacked, after having taken some food or drink, with violent pain, cramp in the stomach, nausea, vomiting, convulsive action, and a sense of suffocation; or, if he is seized, under the same circumstances, with vertigo, giddiness, delirium, or unusual drowsiness. All these symptoms may, however, be the effect of sudden illness, and the examiner should therefore consider the possibility of the existence of some disease which would explain the symptoms. He should inquire into the patient's strength, mode of life, and habit of body, and ascertain whether he had previously complained of ill health, the time at which the noxious substance was taken and the vehicle in which it was given, the taste or odour that was perceived on its administration, and the food or drink that has been lately swallowed, and all subjects that require particular notice.

Taking the mineral acids as the purest examples of poisons that act independently of absorption through the blood, it will be seen on inquiry that all the symptoms they produce, in addition to the direct effects of the local injury, are those of shock, great feebleness, fainting, imperceptible pulse, cold extremities. With respect to the more numerous class which act remotely through the medium of the blood, some certainly possess a very extended influence over the great organs of the body; but the greater number are much more limited in their sphere of action. Some act chiefly by enfeebling or paralysing the heart, others principally by obstructing the pulmonary capillaries, others by obstructing the capillaries of the general system, others by stimulating or depressing the functions of the brain or of the spinal cord.

Till the close of the eighteenth century it was the custom to decide questions of poisoning from the symptoms only. In fact, up to that time no other evidence was accounted so infallible; and for this simple reason, that in reality the other branches of evidence were even more imperfectly understood. As late as 1760 the solemn opinions of whole colleges were sometimes grounded almost exclusively on the symptoms. About that time, however, doubts began to be entertained of the infallibility of such evidence. These doubts have since assumed gradually a more substantial form; and it is now laid down by all authors in medical jurisprudence that the symptoms, however exquisitely developed, can never justify an opinion in favour of more than high probability.

Having formed an opinion that a poisonous substance has been taken, the next question that arises is to what class it belongs. Some of them act instantaneously, and the effects of most of them are in general fully developed within an hour, or little more. But this character is by no means uniform. The most violent may be made to act, so as to bring on their peculiar symptoms slowly, or even by imperceptible degrees. Thus arsenic, which usually causes violent symptoms from the very beginning, may be so administered as to occasion at first nothing more than slight nausea and general feebleness, and afterwards in slow succession its more customary effects. In like manner corrosive sublimate may be given in such a way as to cause at first mild salivation, and finally gangrene of the mouth. Nevertheless, it still remains true, that the effects of poisons for the most part begin suddenly, when the dose is large. This is an important circumstance in regard to certain active poisons. For when it is considered that in criminal cases they are given for the most part in unnecessarily large doses, it follows that, if the effect ascribed to these poisons in such doses have not begun suddenly, the suspicion is probably incorrect. The same remarks may be applied to the sudden termination of the symptoms. Poison is for the most part given criminally in doses so large that it proves rapidly fatal. Yet this is not always the case: the diseased state occasioned by poisons has often been prolonged for several weeks, sometimes for several months; nay, a person may be carried off by a malady the seeds of which have been sown by the operation of poison years before.

We may suppose that an irritant poison has been taken if the patient has observed that the food or drink which was its vehicle had not its ordinary taste; if he has felt a heat, an irritation, or an extraordinary and sudden dryness at the root of the mouth, and œsophagus, with a constriction or sense of strangling in those parts; if this be succeeded by an obstinate anxiety to vomit, and sharp pains in the stomach and intestines; if there be great thirst, copious discharges, by vomiting and by stool, accompanied with tenesmus, and followed by hiccough, by a sense of constriction across the diaphragm, a difficulty of breathing; if there be great pain in the region of the kidneys, followed by strangury; if convulsions, cramp of the hands, trembling of the lips, extinction of the voice, repeated faintings, cold sweats, and a small and irregular pulse be present; and if, in addition to all these, the intellectual faculties remain perfect, until the disease arrives near its fatal termination.

A narcotic poison, on the other hand, produces the following effects: stupor, numbness, a great inclination to sleep, coldness and stiffness of the extremities, a cold sweat of a fœtid or greasy nature, swelling of the neck and face, protrusion of the eye, with a haggard cast of countenance, thickening of the tongue, frequent vertigo, weakened eyesight or objects presented to it in a fantastic manner, coma, delirium, general debility, palpitation of the heart, the pulse at first full and strong but afterwards unequal and intermittent, paralysis of the lower extremities, retraction of the lips, general swelling of the body, and dilatation of the veins. At the conclusion of the disease, slight convulsions and pain are sometimes present. The narcotico-acrid poisons are distinguished by a combination of several of the above symptoms.

In general terms it may be said, in cases where there are no marks of personal violence upon the body of the deceased, and the history of the symptoms can hardly, in the opinion of a competent medical practitioner, explain a sudden and suspicious death as attributable to natural causes, there is reason to suppose that the deceased came to his death either from some accident, whose pathological effects can be shown at the autopsy, or else by means of some poison which has been absorbed. The object of the medical expert here, as in all other attempts upon the health or life of an individual, is to determine in a precise manner the nature of the disease or cause of death; but, in the majority of crimes, the expert who is called upon to investigate these causes may not begin his examination for weeks or months after the commission of the deed, and until suspicions have led to judicial interference; hence the natural difficulties are greatly enhanced. The great difficulties connected with the discovery or determination of the nature of the symptoms which have preceded the death are hampered by the unwillingness and ignorance of those who witnessed the symptoms; and the fact that a great number of poisons cause symptoms that are almost identical in character, and similar to those of many diseases. On the other hand, the symptoms following the absorption of some of the poisons are very similar, and it may be extremely difficult to determine by the history preceding death that a certain drug had been administered.

To the practitioner the diagnosis of a case of poisoning is of great importance, as by mistaking the symptoms produced by a poison for those arising from natural disease, he may omit to employ the remedial measures which have been found efficacious in counteracting its effects, and thus lead to the certain death of the patient. To a medical jurist a correct knowledge of the symptoms furnishes the chief evidence of poisoning in those cases in which persons are charged with the malicious and unlawful administration of poison.

### Arsenic.

The quantity of arsenic that may be present in the contents of the stomach and intestines is very large. It is very common for the defence to insist that, in a case of supposed arsenic poisoning, unless full fatal doses of arsenic are found absorbed in the system, the prosecution has failed to prove the cause of death. When, however, the arsenic extracted is in weighable quantities, and in such a form that it can be isolated and shown to the jury, and when the care shown by the analyst and his previous reputation make it reasonably certain that the arsenic in question came from the cadaver, it is perfectly proper to insist that quite minute quantities of the poison furnish very strong evidence of the cause of death. Indeed, the best experts are agreed that in perfectly well-defined cases of arsenic poisoning it is not only not uncommon to find but small amounts of the poison, but it may easily happen that no arsenic at all may remain in the system. When arsenic is taken into the system through the stomach, but a short time elapses before nature endeavours by constant and thorough vomiting, and subsequently by purging, to expel the poison before too much has entered the circulation. That this is frequently successful is well known from the numerous instances of persons who have taken large doses and recovered without special treatment. The poison that is absorbed enters the portal circulation, and is stopped by the liver, which retains as much of the arsenic as possible. The poison begins to be stored up in the liver probably in a few minutes, if not seconds, after it is absorbed, and gradually increases in quantity for some fifteen or twenty hours. But from the time it enters the system until it is entirely removed, or death intervenes, active agencies are at work to eliminate it from the body. The most important of these is the kidneys, which begin their work almost at once. Besides this the arsenic is excreted through the bile, the skin, and probably, after some little time, at least, through the large intestine. So it is perfectly evident that if the patient does not die for some time the amount of arsenic present in the system must be diminishing every day.

Generally a dose of from one-third to one-half a grain, taken in a soluble form, will produce symptoms of vomiting and other gastric trouble; and from three to four grains of white arsenic are usually enough to produce death. But in certain parts of the world, notably in Lower Austria and in Styria, and also in the Punjab in India, people exist who have trained themselves to eat with impunity, at more or less regular intervals, arsenic in considerable quantities. These facts have been set up by the defences in almost every important arsenic case of late years, to explain the presence of small quantities of arsenic in the body of the victim. A notable example took place in the famous Maybrick trial in Liverpool, 1889. On this occasion, besides several corroborating circumstances, such as a well-proven inducement for the crime, the agreement of the symptoms and post-mortem appearance of the body with those of arsenic poison, and the consensus of all the physicians in attendance on Mr. Maybrick some two days before his death, that he was being poisoned, there were three points of vital importance to the defence which had to be explained in order to save the defendant. These were, first, the presence of arsenic, although in small quantities, in the liver and intestines of Mr. Maybrick; secondly, the presence of arsenic in large quantities and in numerous forms either in Mrs. Maybrick's room or in articles belonging to her, or to which she had free access; thirdly, that after suspicions had been aroused and food proven free from arsenic had been provided for the patient, a nurse saw Mrs. Maybrick enter her husband's room, secretly take a bottle of beef extract from the room, and bring it back and replace it in the room in a few minutes full of arsenic. The defence claimed, first, that Mr. Maybrick was an arsenic eater. This was supposed on some rather slight evidence. Secondly, that Mrs. Maybrick used the various solutions of arsenic, the extracts of arsenical fly-papers, and the packages of rat poison, white arsenic, and the rest which were in her possession as cosmetics. And, thirdly, Mrs. Maybrick stated that, remembering a white powder which her husband was in the habit of using, she had, at his request, put a teaspoonful of it into the beef extract. These explanations were of more effect upon the outside public than on the jury.

